# Supplementary material for: Virulence Characteristics and Molecular Typing of Carbapenem-Resistant ST15 Klebsiella pneumoniae Clinical Isolates, Possessing the K24 Capsular Type
Source: Antibiotics (Basel). 2023 Feb 28;12(3):479. doi: 10.3390/antibiotics12030479 (PMC10044539; doi:10.3390/antibiotics12030479)
Supplement: Supplementary file 1 [file antibiotics-12-00479-s001.zip › Supplementary Table S1..pdf]

Supplementary Table S1. Primers used in this study.

Category	Genes	Primers	Primers sequence (5′ – 3′)	Size (bp)	Annealing temperature (°C)	Ref
**Type 1 fimbriae**	*fimH-1*	fimH-1 FW	ATGAACGCCTGGTCCTTTGC	688	55	El Fertas-Aissani et al., 2013 [21]
		fimH-1 Rev	GCTGAACGCCTATCCCCTGC			
**Type 3 fimbriae**	*mrkD*	mrkD FW	CCACCAACTATTCCCTCGAA	240	60	Abdulhassan et al., 2015 [98]
		mrkD Rev	ATGGAACCCACATCGACATT			
**Adhesion associated genes**	*mrkA*	mrkA FW	CTGCTGCAATGGCAACTGC	807	65	Wilksch et al., 2011 [101]
		mrkA Rev	GGCAGTCAGAAACATCGATGG			
	cf29a	cf29a FW	GACTCTGATTGCACTGGCTGTG	826	65	Chiang et al., 2016 [99]
		cf29a Rev	GTTATAAGTTACTGCCACGTTC			
	mrkJ	mrkJ FW	CGAAGACAACATTTTATCTCGTAATG	717	62	Johnson and Clegg, 2010 [10]
		mrkJ Rev	CAATCAGTGCCAGTTGTTCGAG			
**The activator of the allantoin regulator gene**	*allS*	allS FW	CAGAAGAGCCAGGTCAC	1090	60	Chiang et al., 2016 [99]
		allS Rev	CTGATGTACGACCCGCAG			
**Enterobactin production**	*entB*	entB FW	ATTTCCTCAACTTCTGGGGC	371	57	El Fertas-Aissani et al., 2013 [21]
		entB Rev	AGCATCGGTGGCGGTGGTCA			
**Aerobactin production**	*iutA*	iutA FW	GGCTGGACATCATGGGAACTGG	300	63	El Fertas-Aissani et al., 2013 [21]
		iutA Rev	CGTCGGGAACGGGTAGAATCG			

**Serumresistance-associated outher membrane lipoprotein**	*traT*	traT FW	GGTGTGGTGCGATGAGCACAG	290	63	El Fertas-Aissani et al., 2013 [21]
		traT Rev	CACGGTTCAGCCATCCCTGAG			
**Regulator of mucoid phenotype**	*rmpA*	rmpA FW	ACTGGGCTACCTCTGCTTCA	535	64	El Fertas-Aissani et al., 2013 [21]
		rmpA Rev	CTTGCATGAGCCATCTTTCA			
**Uridine diphosphate galacturonate 4-epidermase gene**	*uge*	uge FW	GATCATCCGGTCTCCCTGTA	534	63	Regué et al., 2004 [100]
		uge Rev	TCTTCACGCCTTCCTTCACT			
**Mucoviscosity-associated gene A**	*magA*	magA FW	GGTGCTCTTTACATCATTGC	1283	60	Chiang et al., 2016 [99]
		magA Rev	GCAATGGCCATTTGCGTTAG			
**K24 capsular serotype**	*wziK24*	wziK24 FW	AGATAATAGGCAACAGCGTTCT	648	62	Own primer
		wziK24 Rev	GATACGTTAAACGCCTCAAGTA			
