# Supplementary material for: Virulence Characteristics and Molecular Typing of Carbapenem-Resistant ST15 Klebsiella pneumoniae Clinical Isolates, Possessing the K24 Capsular Type
Source: Antibiotics (Basel). 2023 Feb 28;12(3):479. doi: 10.3390/antibiotics12030479 (PMC10044539; doi:10.3390/antibiotics12030479)
Supplement: Supplementary file 1 [file antibiotics-12-00479-s001.zip › Supplementary Table S2a and b.pdf]

Supplementary Table S2/a: Antimicrobial susceptibility pattern (EUCAST) of the carbapenem-resistant *K. pneumoniae* isolates (S: susceptible, I: susceptible, increased exposure, and R: resistant) of this study. AMP: Ampicillin, AMC: Amoxicillin/Clavulanic acid, PTZ: Piperacillin/Tazobactam, SAM: Ampicillin/Sulbactam, FEP: Cefepime, CTX: Cefoxitin, CAZ: Ceftazidime, CRO: Cefrtiaxone, CXM: Cefuroxime, FOX: Cefoxitin, ETP: Ertapenem, IMI: Imipenem, MEM: Meropenem, CIP: Ciprofloxacin, LEV: Levofloxacin, AN: Amikacin, GM: Gentamicin, TM: Tobramycin.

Antibiotics (μg)		S (n)	S (%)	I (n)	I (%)	R (n)	R (%)
Penicillins	AMP 10	0	0	0	0	39	100
	AMC 20/10	0	0	0	0	39	100
	PTZ 30/6	0	0	0	0	39	100
	SAM 10/10	0	0	0	0	39	100
Cephalosporins	FEP 30	0	0	0	0	39	100
	CTX 5	0	0	0	0	39	100
	CAZ 10	0	0	0	0	39	100
	CRO 30	0	0	0	0	39	100
	CXM 30	0	0	0	0	39	100
	FOX 30	0	0	0	0	39	100
Carbapenems	ETP 10	0	0	0	0	39	100
	IMI 10	0	0	0	0	39	100
	MEM 10	0	0	0	0	39	100
Fluoroqu-inolones	CIP 5	0	0	0	0	39	100
	LEV 5	0	0	0	0	39	100
Aminoglycosides	AN 30	19	48.71	20	51.28	0	0
	GM 10	12	30.76	27	69.23	0	0
	TM 10	0	0	1	2.56	38	97.43

Supplementary Table S2/b: Minimum Inhibitory Concentration (MIC) for 17 antibiotics (mg/L) against the clinical *K. pneumoniae* isolates (n=39). AMP: Ampicillin, AMC: Amoxicillin/Clavulanic acid, PTZ: Piperacillin/Tazobactam, SAM: Ampicillin/Sulbactam, FEP: Cefepime, CTX: Cefoxitin, CAZ: Ceftazidime, CRO: Cefrtiaxone, CXM: Cefuroxime, FOX: Cefoxitin, ETP: Ertapenem, IMI: Imipenem, MEM: Meropenem, CIP: Ciprofloxacin, LEV: Levofloxacin, AN: Amikacin, GM: Gentamicin, TM: Tobramycin.

**Strain No.**	**Penicillins**				**Cephalosporins**						**Carbapenems**			**Fluoroquinolones**		**Aminoglycosides**		
	**AMP**	**AMC**	**PTZ**	**SAM**	**FEP**	**CTX**	**CAZ**	**CRO**	**CXM**	**FOX**	**ETP**	**IMI**	**MEM**	**CIP**	**LEV**	**AN**	**GM**	**TM**
**10/1**	16	16	32	16	4	4	4	8	8	16	2	8	8	1	2	8	1	8
**10/4**	16	16	32	16	8	4	4	8	8	16	2	8	8	1	2	8	1	8
**10/6**	16	16	32	16	8	4	4	8	8	16	2	8	8	1	4	8	1	8
**11/1**	16	16	32	16	8	4	4	8	8	16	2	8	8	2	4	8	1	8
**11/3**	16	16	32	16	8	2	2	8	8	16	2	8	8	2	4	8	1	8
**53/1**	16	16	32	16	8	2	2	8	8	16	2	8	8	2	4	8	1	8
**53/2**	16	16	32	16	8	2	2	8	8	16	2	8	8	2	4	8	1	8
**53/3**	16	16	32	16	4	2	2	8	8	16	2	8	8	2	4	8	1	8
**53/4**	16	16	32	16	4	2	2	8	8	16	2	8	8	2	4	8	1	8
**53/5**	16	16	16	16	8	2	2	8	8	16	2	8	8	2	2	8	1	8
**53/6**	16	16	16	16	8	2	2	4	8	16	2	8	8	2	2	4	1	8
**53/8**	16	16	16	16	8	4	4	4	8	16	2	8	8	1	2	4	1	8
**53/9**	16	16	16	16	8	4	4	4	8	16	2	8	8	1	2	4	4	8
**53/10**	16	16	16	16	16	4	4	4	8	16	2	8	16	1	2	4	4	8
**53/11**	16	16	16	16	16	4	4	4	8	16	2	8	16	1	2	4	4	8
**53/13**	16	16	32	16	16	4	4	4	8	16	2	8	16	1	2	4	4	8
**50/1**	16	16	32	16	16	4	4	4	8	16	2	8	16	2	2	4	4	8
**50/2**	16	16	32	16	16	4	4	4	8	16	2	8	16	2	2	4	4	8
**50/3**	16	16	32	16	16	4	4	4	8	16	2	8	16	1	2	4	4	8
**I/1**	16	16	32	16	16	4	4	4	8	16	2	8	8	1	2	4	4	8
**49/1**	16	16	32	16	16	2	2	4	8	16	2	8	8	2	2	4	4	8
**49/2**	16	16	32	16	16	2	2	4	8	16	2	8	8	2	2	4	4	8
**49/3**	16	16	32	16	16	2	2	4	8	16	2	8	8	2	2	4	4	8
**C6/14**	8	16	32	16	16	2	2	4	8	16	2	8	8	2	2	4	4	8
**C7/15**	8	8	32	16	16	2	2	4	8	16	2	8	8	2	2	4	4	8
**C8/15**	8	8	32	16	8	2	2	4	8	16	2	8	8	2	2	4	4	8
**C10/15**	8	8	32	16	8	2	2	4	8	16	2	8	8	2	2	4	4	8
**C11/15**	8	8	32	16	8	2	2	4	8	16	2	8	8	2	2	4	4	8
**C12/15**	8	8	32	16	8	2	2	4	8	16	2	8	8	2	2	4	4	8
**C13/15**	8	8	16	16	8	2	2	4	8	16	2	8	8	2	2	8	4	8
**C14/15**	16	8	16	16	8	2	2	4	8	16	2	8	8	2	2	8	4	8
**C15/15**	16	8	16	16	8	2	2	4	8	16	2	8	8	2	2	8	4	8
**C16/15**	16	8	16	16	8	2	2	4	8	16	2	8	8	2	2	8	4	8
**C17/15**	16	8	16	16	8	4	4	4	8	16	2	8	8	2	2	8	4	8
**C18/15**	16	8	16	16	8	4	4	4	8	16	2	8	8	2	2	8	4	8
**C1/16**	16	8	16	16	8	4	4	4	8	16	2	8	8	2	2	8	4	8
**C2/17**	16	8	16	16	8	4	4	4	8	16	2	8	8	1	2	8	4	8
**C3/17**	16	8	16	16	8	4	4	4	8	16	2	8	8	1	2	8	4	8
