# Supplementary material for: Virulence Characteristics and Molecular Typing of Carbapenem-Resistant ST15 Klebsiella pneumoniae Clinical Isolates, Possessing the K24 Capsular Type
Source: Antibiotics (Basel). 2023 Feb 28;12(3):479. doi: 10.3390/antibiotics12030479 (PMC10044539; doi:10.3390/antibiotics12030479)
Supplement: Supplementary file 1 [file antibiotics-12-00479-s001.zip › Supplementary Table S3..pdf]

Supplementary Table S3. PFGE based virulence analysis of *K. pneumoniae* isolates.

Cluster I

	Date of isolation	Isolated from	Sequence Type	PCR amplification												Phenotypic tests							Invasion assay (CFU/ml)	
				fimH-1	*mrkD*	*mrkA*	*mrkJ*	*cf29a*	*allS*	*entB*	*iutA*	*traT*	*rmpA*	*uge*	*wziK 24*	Type 1 fimbriae	Type 3 fimbriae	Biofilm produc-tion	Entero-bactin	Aero-bactin	Serum resis-tance	HMV	INT 407	T24
**49/1**	2011	fecal	ST 15	+	+	+	+	–	+	+	–	–	–	+	+	+	+	high	+	–	–	–	100	100
**49/3**	2011	fecal	ST 15	+	+	+	+	–	+	+	–	–	–	+	+	+	+	high	+	–	–	–	0	300
**50/3**	2011	fecal	ST 15	+	+	+	+	–	+	+	–	+	+	+	+	+	+	high	+	–	+	+	0	0
**10/1**	2010	urine	ST 15	+	+	+	+	–	+	+	–	–	–	+	+	+	+	high	+	–	–	–	0	200
**C17/15**	2015	fecal	ST 15	+	+	+	+	–	+	+	–	+	–	+	+	+	+	high	+	–	+	–	0	0
**11/1**	2010	urine	ST 15	+	+	+	+	–	+	+	–	+	+	+	+	+	+	high	+	–	+	+	0	100
**C1/16**	2016	fecal	ST 15	+	+	+	+	–	+	–	–	–	–	+	+	+	+	high	–	–	–	–	0	100
**C14/15**	2015	urine	ST 15	+	+	+	+	–	+	+	–	–	–	+	+	+	+	high	+	–	–	–	500	200
**C15/15**	2015	urine	ST 15	+	+	+	+	–	+	+	–	+	–	+	+	+	+	high	+	–	+	–	300	200
**10/6**	2010	urine	ST 15	+	+	+	+	–	+	+	–	–	–	+	+	+	+	high	+	–	–	–	0	900
**I/1**	2011	fecal	ST 15	+	+	+	+	–	+	+	–	–	–	+	+	+	+	high	+	–	–	–	0	100
**53/1**	2011	sputum	ST 15	+	+	+	+	–	+	+	+	+	–	+	+	+	+	high	+	+	+	–	100	300
**C12/15**	2015	urine	ST 15	+	+	+	+	–	+	+	–	+	–	+	+	+	+	high	+	–	+	–	400	900

Cluster II

	Date of isolation	Isolated from	Sequence Type	PCR amplification												Phenotypic tests							Invasion assay (CFU/ml)	
				fimH-1	*mrkD*	*mrkA*	*mrkJ*	*cf29a*	*allS*	*entB*	*iutA*	*traT*	*rmpA*	*uge*	*wzi K24*	Type 1 fimbriae	Type 3 fimbriae	Biofilm produc-tion	Entero-bactin	Aero-bactin	Serum resis-tance	HMV	INT 407	T24
**50/1**	2011	blood culture	ST 15	+	+	+	+	–	+	+	–	–	–	+	+	+	+	high	+	+	+	–	100	300
**53/11**	2011	urine	ST 15	+	+	+	+	–	+	+	–	+	+	+	+	+	+	high	+	–	+	+	0	200
**53/3**	2011	urine	ST 15	+	+	+	+	–	+	+	–	+	+	+	+	+	+	high	+	–	+	+	300	200
**53/4**	2011	sputum	ST 15	+	–	–	–	–	+	+	–	+	–	+	+	+	–	poor	+	–	+	–	0	300
**49/2**	2011	fecal	ST 15	+	–	–	–	–	+	–	–	+	–	+	+	+	–	poor	–	–	+	–	0	500
**50/2**	2011	sputum	ST 15	+	+	+	+	–	+	+	–	–	–	+	+	+	+	high	+	–	–	–	100	100
**53/2**	2011	sputum	ST 15	+	+	+	+	–	+	+	–	–	–	+	+	+	+	high	+	–	–	–	0	500
**C13/15**	2015	fecal	ST 15	+	+	+	+	–	+	+	–	–	–	+	+	+	+	high	+	–	+	–	0	100

**53/5**	2011	blood culture	ST 15	+	+	+	+	–	+	+	–	+	–	+	+	+	+	high	+	–	+	–	0	800
**53/6**	2011	fecal	ST 15	+	+	+	+	–	+	+	–	–	–	+	+	+	+	high	+	–	–	–	100	800
**53/9**	2011	fecal	ST 15	+	+	+	+	–	+	+	–	–	–	+	+	+	+	high	+	–	–	–	0	400
**53/13**	2011	fecal	ST 15	+	+	+	+	–	+	+	–	–	–	+	+	+	+	high	+	–	–	–	0	100
**53/8**	2011	urine	ST 15	+	+	+	+	–	+	+	–	–	–	+	+	+	+	high	+	–	–	–	0	200

Cluster III

	**Date of isolation**	**Isolated from**	**Sequence Type**	**PCR amplification**												**Phenotypic tests**							**Invasion assay (CFU/ml)**	
				fimH-1	***mrkD***	***mrkA***	***mrkJ***	***cf29a***	***allS***	***entB***	***iutA***	***traT***	***rmpA***	***uge***	***wzi K24***	**Type 1 fimbriae**	**Type 3 fimbriae**	**Biofilm produc-tion**	**Entero-bactin**	**Aero-bactin**	**Serum resis-tance**	**HMV**	**INT 407**	**T24**
**C16/15**	2015	fecal	ST 15	+	+	+	+	+	+	+	–	+	–	+	+	+	+	high	+	–	+	–	0	0
**C7/15**	2015	fecal	ST 15	+	+	+	+	+	+	+	–	+	–	+	+	+	+	high	+	–	+	–	0	0
**C11/15**	2015	fecal	ST 15	+	+	+	+	+	+	+	–	+	–	+	+	+	+	high	+	–	+	–	0	0
