# Supplementary material for: Virulence Characteristics and Molecular Typing of Carbapenem-Resistant ST15 Klebsiella pneumoniae Clinical Isolates, Possessing the K24 Capsular Type
Source: Antibiotics (Basel). 2023 Feb 28;12(3):479. doi: 10.3390/antibiotics12030479 (PMC10044539; doi:10.3390/antibiotics12030479)
Supplement: Supplementary file 1 [file antibiotics-12-00479-s001.zip › Supplementary Table S4..pdf]

Supplementary Table S4. Comparative pathogenomics of *K. pneumoniae* strain 11/3, strain 50/1, strain 53/2 and strain 53/2 with VFDB database.

**Virulence genes**	**Function**	**Localization**				**Length**	**Ref.**
		**11/3** (fecal)	**50/1** (blood culture)	**53/2** (sputum)	**53/3** (urine)		
acrA	acriflavine resistance protein A	687 894-689 087		166 313-167 506	263 360-264 553	1194	*K. pneumoniae* NTUH-K2044
acrB	acriflavine resistance protein B	211 419-212 970	535 254-538 400	167 529-170 675	1 492-4 449	3147	*K. pneumoniae* NTUH-K2044
clpV/tssH	type VI secretion system ATP-ase TssH	164 039-166 694			508 276-510 930	2460	*K. pneumoniae* HS11286
cpsACP	phosphatase PAP2 family protein				8 944-9 572	630	*K. pneumoniae* NTUH-K2044
dotU/tssL	type VI secretion system protein, DotU/TssL family	420 377-421 066	6 904-7 593		505 123-505 812	690	*K. pneumoniae* HS11286
entE	enterobactin synthase subunit E	553 766-555 373	344 618-346 225		22 139-23 746	1608	*K. pneumoniae* NTUH-K2044
entF	enterobactin synthase subunit F	562 522-566 403	353 374-357 255	176 758-180 639	11 109-14 990	3882	*K. pneumoniae* NTUH-K2044
exeF	general secretion pathway protein F	67 830-68 809	294 300-295 279		293 779-294 758	1221	*Aeromonas hydrophila* ATCC 7966
fepA	outer membrane receptor FepA	568 113-570 341	358 965-361 193	172 820-175 048	7 171-9 399	2229	*K. pneumoniae* NTUH-K2044
fes	enterobactin/ferric enterobactin esterase	566 645-567 853			9 659-10 867	1209	*K. pneumoniae* NTUH-K2044
fimD	outer membrane usher protein, Type I fimbriae	166 716-169 361	255 581-258 226	224 136-226 721	175 190-177 835	2646	*K. pneumoniae* NTUH-K2044
fimH	type I fimbrial adhesin precursor, type I fimbriae		253 607-254 515	227 787-228 695	178 901-179 800	909	*K. pneumoniae* NTUH-K2044
fimK	transcriptional regulator, Type I fimbriae		252 195-253 439	228 863-230 104	179 977-181 218	1242	*K. pneumoniae* NTUH-K2044
**Virulence genes**	Function	Localization				Length	Ref.
		**11/3** (fecal)	**50/1** (blood culture)	**53/2** (sputum)	**53/3** (urine)		
fyuA	pesticin/yersiniabactin receptor protein		82 340-84 361	611 454-613 475	401 682-403 703	2022	*Yersinia pestis* C092

galF	UTP-glucose-1-phosphate uridyltransferase subunit GalF				9 965-10 861	897	*K. pneumoniae* NTUH-K2044
icmF/tssM	type VI secretion protein TssM	11 335-14 416	7 851-10 932	1 093 627-1 096 708	3 127-6 208	3426	*K. pneumoniae* HS11286
impA/tssA	type VI secretion system protein TssA		10 934-11 154		6 210-6430	408	*K. pneumoniae* HS11286
iroE	siderophore esterase IroE	5 265-6 200	74 231-75 166		74 293-75 228	936	*K. pneumoniae* NTUH-K2044
irp1	yersiniabactin biosynthetic protein Irp1		87 970-97 461	598 354-607 845	388 582-398 073	9492	*Yersinia pestis* C092
irp1/ybt	yersiniabactin polyketide synthase HMWP1				388 582-398 073	9492	*K. pneumoniae* NTUH-K2044
irp2	yersiniabactin biosynthetic protein Irp2		97 549-103 656	592 159-598 266	382 387-388 494	6108	*Yersinia pestis* C092
irp2/ybt	yersiniabactin non-ribosomal peptide synthetase HMWP2				382 387-388 494	6108	*K. pneumoniae* NTUH-K2044
manB	phosphomannomutase	551 716-553 086				1371	*K. pneumoniae* NTUH-K2044
manC	mannose-1-phosphate guanylyltarnsferase	550 278-551 693				1416	*K. pneumoniae* NTUH-K2044
mrkB	fimbrial chaperone protein mrkB precursor, Type III fimbriae		7 086-7 787			702	*K. pneumoniae* NTUH-K2044
mrkC	fimbrial biogenesis outer membrane usher protein MrkC percursor, Type III fimbriae	179 561-182 047	268 426-270 912	211 390-213 876	162 504-164 990	2487	*K. pneumoniae* NTUH-K2044
mrkD	fimbrial adhesin protein percursor MrkD, Type III fimbriae		270 903-271 898	210 404-211 399	161 518-162 513	996	*K. pneumoniae* NTUH-K2044
mrkH	transcriptional activator, Type III fimbriae		13 405-14 115			711	*K. pneumoniae* NTUH-K2044
mrkJ	phosphodiesterase, Type II fimbriae		11 955-12 671			717	*K. pneumoniae* NTUH-K2044
rcsB	transcriptionalregulator RcsB	38 532-39 182	296 931-297 581		297 272-297 922	651	*K. pneumoniae* NTUH-K2044
**Virulence genes**	Function	Localization				Length	Ref.
		**11/3** (fecal)	**50/1** (blood culture)	**53/2** (sputum)	**53/3** (urine)		

sciN/tssJ	type VI secretion system lipoprotein TssJ	20 428-20 970	16 944-17 486		12 220-12 762	543	*K. pneumoniae* HS11286
tssF	type VI secretion system baseplate subunit TssF	17 647-19 401	14 163-15 917	1 099 999-1 101 693	9 439-11 193	1755	*K. pneumoniae* HS11286
tssG	type VI secretion system baseplate subunit TssG	19 365-20 450	15 881-16 966		11 157-12 242	1086	*K. pneumoniae* HS11286
vasE/tssK	type VI secretion system baseplate subunit TssK	419 037-420 380	7 590-8 933		503 783-505 126	1344	*K. pneumoniae* HS11286
vgrG/tssI	type VI secretion system tip protein VgrG	426 186-427 909	61-1 784		510 932-512 655	2370	*K. pneumoniae* HS11286
vipB/tssC	type VI secretion system contractile sheat large subunit VipB	417 483-419 027	8 943-10 487		502 229-503 773	1545	*K. pneumoniae* HS11286
waaF	heptosyltransferase I, LPS		297 826-297 991	204 443-204 608	203 601-203 766	1038	*Pseudomonas aeruginosa* PA01
wbbM	glycosyltransferase, LPS	558 938-560 833				1896	*K. pneumoniae* NTUH-K2044
wzi	surface assembly of capsule				6 550-8 064	1515	*K. pneumoniae* NTUH-K2044
yagW/ecpD	polymerized tip adhesin of ECP fibers	533 138-534 781		320 619-322 262	108 604-110 247	1644	*E. coli* O157:H7
yagX/ecpC	*E. coli* common pilus usher EcpC	530 623-533 148	689 977-692 502	322 252-324 777	106 089-108 614	2526	*E. coli* O157:H7
yagY/ecpB	*E. coli* common pilus chaperone EcpB	529 929-530 597		324 803-325 471	105 395-106 063	669	*E. coli* O157:H7
ybdA	enterobactin exporter EntS	558 316-559 557			17 955-19 196	1242	*K. pneumoniae* NTUH-K2044
